# Annual Wellness Visits and Early Dementia Diagnosis Among Older Adults Receiving Medicare Benefits

**DOI:** 10.1002/alz.094615

**Published:** 2025-01-09

**Authors:** Huey‐Ming Tzeng, Mukaila A Raji, Yong Shan, Peter Cram, Yong‐ Fang Kuo

**Affiliations:** ^1^ University of Texas Medical Branch, Galveston, TX USA; ^2^ University of Toronto, Toronto, ON Canada

## Abstract

**Background:**

Early recognition of cognitive impairment (CI) and timely diagnoses of mild CI (MCI) and Alzheimer’s Disease and Related Dementia (ADRD) are key to optimal dementia care. No previous research has examined the effects of Medicare annual wellness visits (AWVs) on early CI recognition among Medicare beneficiaries. Here, we assessed the association between incident diagnosis at MCI, moderate or severe ADRD stages in 2017‐2020, and having a Medicare AWV in the three years prior to diagnosis.

**Method:**

This population‐based, case‐control study used 100% Texas fee‐for‐service Medicare data from 2014 to 2020; population: Medicare beneficiaries diagnosed with incident MCI or ADRD from 2017 to 2020. The exposure is Medicare AWVs. The primary outcome is MCI, moderate or severe ADRD status by ICD‐9‐CM and ICD‐10‐CM codes. We performed a multinomial logistic regression model to examine the association.

**Result:**

Medicare beneficiaries (n = 197,356) who had an AWV were 23‐66% more likely to be diagnosed at the MCI stage and 8‐30% more likely to be diagnosed at the moderate ADRD stage compared to diagnoses at the severe ADRD stage relative to those who did not have an AWV. There was a dose‐response relationship; as the number of AWV visits in the three years increased, the magnitude of the likelihood of early diagnosis increased.

**Conclusion:**

This study suggests that receipt of a Medicare AWV is associated with increased identification of early CI.

